# Enhancing Patient Safety: A Cross-Sectional Study to Assess Medical Interns’ Attitude and Knowledge About Medication Safety in Saudi Arabia

**DOI:** 10.7759/cureus.50505

**Published:** 2023-12-14

**Authors:** Raghad Hijazi, Hatouf Sukkarieh, Rami Bustami, Jibran Khan, Reema Aldhalaan

**Affiliations:** 1 College of Medicine, Alfaisal University, Riyadh, SAU; 2 College of Business, Alfaisal University, Riyadh, SAU

**Keywords:** patient safety, knowledge, safety reporting system (srs) awareness, interns, medication errors

## Abstract

Introduction and aim

Medication errors (MEs) pose a severe threat in the medical field. Since such errors are preventable, it is paramount for all healthcare workers to be educated on the matter. This study aimed to assess medical interns’ attitudes and knowledge of medication safety and errors. We also aimed to validate current university programs to educate students about medication safety and errors.

Methods

A cross-sectional study that utilized a self-administered online questionnaire comprised 31 questions. The questionnaire was distributed via social media networks, such as WhatsApp, Twitter, email, Instagram, and Snapchat among 100 medical, pharmacy, and nursing interns in Saudi Arabia. The study population included both Saudi and non-Saudi interns.

Results

The majority of participants, comprising 92% (n=92), indicated that they were familiar with the definition of medication errors (ME). Additionally, 85% (n=85) expressed their willingness to report instances of MEs when medications were not prescribed but required. Moreover, 90% (n=90) of the surveyed individuals expressed their willingness to report MEs in situations where patients did not receive medications as prescribed. In cases where patients experienced harm and required treatment due to an ME, 91% (n=91) of respondents committed to reporting such incidents. A total of 52 (52%) respondents stated that they would report MEs regardless of whether they reached/harmed the patient. A good ME knowledge level was observed in 48% of respondents. A higher likelihood of good ME knowledge was significantly associated with safety reporting system (SRS) awareness and reporting MEs regardless of whether they reached/harmed the patient (p<0.05). College, awareness/attitude, or other demographic factors were not significantly related to ME knowledge (p>0.05).

Conclusion

This study showed that although interns in the healthcare field do have some knowledge about MEs, there is still a significant need to improve their knowledge. This can be achieved through various ways, one of which is by implementing this topic into the university curricula.

## Introduction

Medication errors (MEs) pose a serious burden on patients, affecting their health and economic status. To date, there is no unified definition of MEs; a systematic review that assessed MEs in the Middle East found that of the 50 studies used, 17 had different definitions [1]. The most accurate is “A medication error is any preventable event that can lead to or cause inappropriate medication use or patient harm when the medication is in the control of the health care professional, patient, or consumer” [2]. MEs can occur at various stages, including prescription, dispensing, administration, and monitoring.

Medication errors are ranked as the eighth leading cause of preventable deaths in the United States, resulting in up to 225,000 deaths per year [3]. Furthermore, MEs have had a severe economic burden, costing 3.5 billion dollars annually in American hospitals [4]. A systematic review conducted in Africa that assessed MEs and adverse drug events in hospitals concluded that MEs are common. In two of the included studies, the incidence of MEs was reported to be 7.5-7.8 per 100 admissions [5]. It can be concluded that MEs are a problem in many world regions and, therefore, need to be adequately addressed.

These errors have had tremendous impacts on patients’ lives throughout the years. A devastating and easily preventable incident was reported in the United Kingdom; a day of great tragedy in 2004 recorded the death of a 30-year-old nurse who died two hours after giving birth to her son due to the administration of a higher dose than needed of bupivacaine [6]. Such a preventable mistake is one incident of many. In fact, an economic analysis conducted in the United Kingdom found that of the 237 million MEs conducted annually, 66 million were potentially preventable [7].

The causes of MEs are multifactorial. A retrospective study conducted in a tertiary care hospital in the Kingdom of Saudi Arabia (KSA) found the three top reasons for MEs were lack of staff education (67.47%, n=7,208), miscommunication of drug orders (19.04%, n=2,034), and look-alike/sound-like medication problems (7.54%, n=805) [8]. In addition, a nationwide observational study conducted in the KSA showed that the top two contributing factors to MEs were healthcare professionals’ work overload (31.6%, n=22,541) and lack of experience (22.7%, n=16,192) [9]. Other factors, such as increased burnout, were contributing factors. Similarly, a study that analyzed the association between physician burnout and medical errors concluded that each standard deviation-unit increase in burnout was associated with increased self-reported medical errors [10].

Furthermore, MEs can occur at various stages of the medication process, including prescribing, dispensing, administering, and monitoring. A study in Qatar investigated MEs' incidence, nature, and severity. It included 5,103 reports submitted between 2015-2017. When assessing the stage at which the ME occurred, first were prescribing errors (87.9%, n=4,485), then administration errors (6.3%, n=322), followed by dispensing errors (5.1%, n=260), and lastly, monitoring errors (0.7%, n=36) [11]. To overcome such problems, tools such as Lean Six Sigma are used (Lean Six Sigma is a globally proven methodology for the sustainable and demonstrable improvement of processes and organizations) [12]. This process improvement methodology can reduce MEs' operational costs and increase patient safety [13]. This tool was utilized in a study conducted in an inpatient pharmacy of a teaching hospital in Thailand. They used the Lean Six Sigma methodology to reduce MEs. Their results showed a reduction of 66.6% in dispensing errors [14].

While there is existing literature on the knowledge and attitudes of healthcare practitioners in Saudi Arabia regarding medication errors (MEs), there is a need for more research focusing on the undergraduate population and interns. This study aimed to fill this gap. The present study aimed to assess healthcare interns' (medical, nursing, and pharmacy) attitudes and knowledge toward medication safety and to validate current university programs set to educate students about medication safety and errors through a self-administered survey.

## Materials and methods

A questionnaire was developed and administered to medical, pharmacy, and nursing interns in various universities in Saudi Arabia (Alfaisal University, King Saud University, King Saud Bin Abdulaziz University for Health Sciences, Imam Muhammad Ibn Saud Islamic University, Almaarefa University, Dar Al Uloom University, Alfarabi Colleges, University of Hail, and various other universities) to study and assess their attitudes and knowledge of medication safety and errors.

The questionnaire consisted of 31 items and was divided into four sections as follows: demographics (gender, age, nationality, marital status, employment status), knowledge of MEs and the reporting system, participants’ attitudes, and universities’ current curricula on MEs and safety. Knowledge, attitudes, and safety were measured at a five-point Likert scale as follows: strongly disagree, disagree, neutral, agree, and strongly agree.

Participants were provided with an electronic copy of the questionnaire and were given enough time to complete it (self-answered). The completed questionnaires were electronically collected and safely stored. The data were uploaded and saved into an appropriately designed Excel spreadsheet.

All participants in the study fulfilled the specified criteria, having obtained their undergraduate degree from a Saudi Arabian college in one of the following disciplines: medicine, nursing, or pharmacy. Moreover, they completed their clinical rotations at hospitals situated in Saudi Arabia.

Validity and reliability

The responses were processed in accordance with the best practices for raw data management to identify any inaccuracies or incompleteness before the statistical analyses. Responses to all items in the questionnaire were checked and compared against the possible minimum and maximum values of each variable, and items with implausible values were flagged. A similar process was applied to demographic variables by running general frequency analyses to identify potential anomalies.

Statistical analysis

Descriptive statistical analyses were performed on the data for the study participants. Continuous variables were summarized using mean and standard deviation (SD), median, and IQR, and proportions were used for nominal and ordinal variables. Knowledge of medication errors, attitudes, and safety was analyzed and compared by demographic characteristics. Comparisons were made using the chi-square test or ANOVA. Knowledge scores were also evaluated and compared by demographic characteristics using the t-test/Mann-Whitney U test or the Kruskal-Wallis test, as appropriate. The highest score achieved by any participant could be 18. The correct response was awarded a score of 1, while an incorrect response was given a score of 0. The knowledge score was calculated as the percentage of correctly answered knowledge questions (range: 0-100), with higher scores indicating better knowledge. Good ME knowledge was indicated by a score of 70 or higher. A logistic regression model was utilized to examine the impact of participants’ demographics on ME knowledge. Statistical significance was considered at p<0.05. All statistical analyses were performed using IBM SPSS 29.0 (Armonk, NY: IBM Corp).

Ethical considerations

Institutional review board (IRB) approval for the study was obtained from the Institutional Review Board of Alfaisal University. Informed consent was obtained from all study participants. Participant confidentiality was maintained throughout the study as no personal identifiers were gathered.

## Results

A total of 100 participants completed the questionnaire (95%). The content validity of the questionnaire items measuring attitudes and knowledge about MEs was established by two experts who examined the appropriateness of the content after making necessary modifications to items to ensure they were comprehensive and accurately assessed and measured. In addition, the reliability of the questionnaire was examined using Cronbach’s alpha (α), which is a measure of internal consistency, indicating how closely related a set of items is as a group. The Cronbach’s α value was 0.72, indicating an acceptable level of internal consistency.

Table 1 shows descriptive statistics for respondents’ demographic characteristics. The majority of respondents were females (72%, n=72), aged 18-25 years (84%, n=84), and from Alfaisal University (65%, n=65). Also, most respondents were single (92%, n=92), non-Saudi (77%, n=77), and not employed (85%, n=85).

**Table 1:** Study population demographics demonstrated in percentage and number. Total number of respondents is 100. The data are presented as n (%).

Characteristic		n	Percentage
Gender	Male	28	28.0%
	Female	72	72.0%
Age (years)	18-25	84	84.0%
	26 or older	16	16.0%
Nationality	Saudi	58	58.0%
	Non-Saudi	42	42.0%
Marital status	Single	92	92.0%
	Married	8	8.0%
Employment status	Employed	15	15.0%
	Not employed	85	85.0%
College	Medicine	71	71.0%
	Pharmacy	23	23.0%
	Nursing	6	6.0%

The vast majority of respondents (92%, n=92) reported awareness of the definition of ME and would report ME when a medication is not prescribed (90%, n=90), when a patient is not receiving a medication as prescribed (85%, n=85), or when a patient is harmed and requires subsequent treatment for the consequences of the ME (91%, n=91) (Table 2). Only 60% (n=60) stated that they would report MEs if preventable before reaching the patient. A total of 40% (n=40) of respondents reported that they fear being blamed/punished for reporting MEs, 26% (n=26) preferred educating people rather than reporting MEs, 55% (n=55) stated that their university curriculum explains the importance of MEs, while only 26% (n=26) reported that their curriculum explains what ME reporting is. Furthermore, only 40% (n=40) said their curriculum includes enough medication safety sessions.

**Table 2:** Awareness and attitudes toward medication safety (Likert items). Total number of respondents are 100. The data are presented as mean±standard deviation (SD). ME: medication error

Item	Mean	SD	Percent agree/strongly agree
Awareness of the definition of ME	4.40	0.72	92.0%
Not my responsibility to report ME	1.72	0.92	5.00%
Would report ME, prevented before reaching the patient	3.65	1.04	60.0%
Would report ME, medication not prescribed for patient	4.41	0.87	90.0%
Would report ME, patient harmed and required treatment	4.51	0.83	91.0%
Would report ME, patient not receiving medication as prescribed	4.27	0.94	85.0%
Would not report ME, patient prescribed an inadequate dose	2.61	1.38	31.0%
Prefer educating people rather than reporting ME	2.75	1.10	26.0%
Fear of being blamed/punished for reporting ME	2.99	1.28	40.0%
Curriculum includes enough sessions on medication safety and ME	3.41	1.41	40.0%
Curriculum explains importance of ME reporting	3.84	1.39	55.0%
Curriculum explains how to report ME	2.98	1.38	26.0%

Good ME knowledge, as indicated by a score of 70 (out of 100) or higher, was observed in 48% (n=48) of respondents. Significantly different ME knowledge levels were observed by respondents’ demographic characteristics and their awareness and attitudes toward medication safety (Table 3). Pharmacy students had substantially better ME knowledge than those from other colleges (69.6%, n=70 vs. 43.7%, n=44, 16.7%, n=17; p=0.028). Significantly better ME knowledge was also observed for those reporting safety reporting system (SRS) awareness (66.7%, n=67 vs. 30.8%, n=31; p<0.001). Furthermore, significantly better ME knowledge was also observed for respondents reporting that their university curriculum includes ME (53.2%, n=53 vs. 28.6%, n=29; p=0.045), includes adequate ME sessions (53.2%, n=53 vs. 40%, n=40; p=0.050), explains importance of ME reporting (58.2%, n=58 vs. 35.6%, n=36; p=0.024), and explains how to report MEs (65.4%, n=65 vs. 41.9%, n=42; p=0.039).

**Table 3:** Good ME knowledge by demographic factors and awareness, attitudes toward medication safety. *Good ME knowledge is indicated by a knowledge score of 70 or higher out of 100. **Based on the chi-square test for testing the hypothesis of the association between demographic factors, awareness, and attitudes toward medication safety, and an indicator for good ME knowledge. Total number of respondents is 100. The p-value <0.05 is considered significant. The data are presented as n (%). ME: medication error; SRS: safety reporting system

Factor		n	Good ME knowledge*	Percentage	p-Value**
Demographic factors					
College	Medicine	71	31	43.7%	0.028
	Pharmacy	23	16	69.6%	
	Nursing	6	1	16.7%	
Age (years)	18-25	84	42	50.0%	0.359
	26 or older	16	6	37.5%	
Awareness, attitudes toward medication safety					
SRS awareness	Yes	48	32	66.7%	<0.001
	No	52	16	30.8%	
Curriculum includes ME	Yes	79	42	53.2%	0.045
	No	21	6	28.6%	
Curriculum includes enough ME sessions	Yes	40	24	60.0%	0.050
	No	60	24	40.0	
Curriculum explains ME reporting importance	Yes	55	32	58.2%	0.024
	No	45	16	35.6%	
Curriculum explains ME reporting	Yes	26	17	65.4%	0.039
	No	74	31	41.9%	

Results from logistic regression analysis showed that a higher likelihood of good ME knowledge was significantly associated with SRS awareness (OR=4.07, p=0.008) (Table 4). In addition, those respondents who stated that they would report ME regardless of whether it reached/harmed the patient had a significantly higher likelihood of good ME knowledge (OR=2.52, p=0.037). College, other demographics, and awareness/attitude factors were not significantly related to ME knowledge (p>0.05).

**Table 4:** Multivariate logistic regression model for good ME knowledge. Total number of respondents is 100. The p-value <0.05 is considered significant. The data are presented as percentages. ME: medication error; SRS: safety reporting system

Factor		Percent	OR	95% CI	p-Value
College	Medicine/nursing	29.0%	1.00	Ref.	0.831
	Pharmacy	71.0%	1.15	0.33-3.95	
SRS awareness	No	52.0%	1.00	Ref.	0.008
	Yes	48.0%	4.07	1.45-11.43	
Reporting ME regardless	No	48.0%	1.00	Ref.	0.037
	Yes	52.0%	2.52	1.06-5.98	

## Discussion

In the United Kingdom, almost £1.1 billion yearly is spent on preventable MEs. Furthermore, in Saudi Arabia, one-fifth of the errors encountered in primary care settings are due to MEs. This is just a marginal representation of the burden such errors pose. Overall, through the carefully curated survey, we found that our study population had sufficient awareness of the general definition of MEs and when to report such errors (table in the appendix). Most respondents (92%, n=92) reported awareness of the meaning of MEs, would report MEs when a medication was not prescribed (90%, n=90), when the patient was not receiving a medication as prescribed (85%, n=85), or when the patient was harmed and required treatment (91%, n=91). The aforementioned findings are similar to the findings of a study that assessed the knowledge and attitudes of healthcare practitioners about MEs, around 90% (n=328) demonstrated a good attitude towards reporting MEs [15]. This highlights that current university curricula provide students with the foundational exposure needed for their future practice. Moreover, our study revealed that when reporting near-miss situations, merely 60% (n=60) of participants indicated their intention to report the error, even in cases where preventive measures were effectively dealt with before reaching the patient.

When looking at the respondents' attitudes towards MEs, a high percentage of 40% (n=40) feared the consequences of reporting MEs. Interestingly, those respondents who stated that they would report ME regardless of whether it reached/harmed the patient had a significantly higher likelihood of good ME knowledge (OR=2.52, p=0.037). This is similar to the findings reported by a Brisbane study that assessed final-year medical students' attitudes towards medication safety. They found that 40% (n=40) of the students believed if they were to report an error, it would not be dealt with constructively [16].

Furthermore, it was concluded that 26% (n=26) of the interns preferred educating individuals rather than reporting MEs, this is complementary to what Alsulami et al. found where 21.6% (n=77) of their participants chose to educate committers of MEs rather than report the mistakes [15]. They concluded that having a blame-free learning culture at the unit and organizational levels, accompanied by anonymous, efficient reporting systems and supportive management behavior for healthcare professionals, could help reduce hindrances to reporting MEs [17].

Familiarizing future healthcare practitioners with high-risk medications enhances medication safety and prevents adverse effects. When asked to identify high-risk medications, only 67% (n=67) of our respondents correctly identified them. This is concerning because a lack of awareness about the potential dangers of these high-risk medications can lead to fatal consequences. A randomized controlled trial assessed nurses' knowledge of high-alert medications (HAM) by comparing their knowledge about HAM before and after taking an educational intervention PowerPoint. Results showed an 18.9% increase post-intervention in the intervention group (n=113) (pre-test vs. post-test: 77.2±15.5 vs. 94.7±7.6) [18]. This again highlights the importance of implementing educational programs about medication safety.

When analyzing pharmacy students' knowledge, respondents from the College of Pharmacy had significantly better ME knowledge than those in other colleges (69.6%, n=70 vs. 43.7%, n=44, and 16.7%, n=18; p=0.028). This aligns with the findings of Warholak et al., who discovered a significantly higher error identification rate in pharmacy students than in nursing and medical students [19]. Khan et al. concluded that final-year pharmacy students demonstrated a higher level of understanding regarding adverse drug reactions (ADRs) and expressed more favorable attitudes toward their ability to manage and report them than final-year medical students. These studies illustrate the role of the increased number of pharmacology and pharmacotherapeutics course hours completed by pharmacy students [20].

Significantly better ME knowledge was observed for those reporting SRS awareness (66.7%, n=67 vs. 30.8%, n=31; p<0.001). Additionally, our results revealed that a higher likelihood of good ME knowledge was significantly associated with SRS awareness (OR=4.07, p=0.008). Better ME knowledge was also noted for respondents reporting that their university curriculum includes ME (53.2%, n=53 vs. 28.6%, n=29; p=0.045), includes adequate ME sessions (53.2% n=53 vs. 40%, n=40; p=0.050), explains the importance of ME reporting (58.2%, n=58 vs. 35.6%, n= 36; p=0.024), and explains how to report ME (65.4%, n=65 vs. 41.9%, n=42; p=0.039). A North American study by Karpa et al. aimed to assess the effectiveness of implementing a medication safety curriculum; the curriculum included both theoretical (lectures and assignments) and practical (encounters with patients) training, with the administration of pre-program and post-program tests to assess the program's effectiveness. The enrolled students were able to transfer their theoretical skills to patient care. The enrolled students identified an average of 2.5 medication-related problems on average per patient from a panel of individuals who had recently transitioned from hospital to home [21]. This further highlights the importance of enhancing students' knowledge about the subject.

In contrast, overall good ME knowledge was observed in only 48% (n=48) of our respondents. This is analogous to a study conducted across European universities, which showed that final-year medical students had inadequate clinical pharmacology and therapeutics knowledge, suggesting that their core curriculum should be reviewed and updated to enhance learning [22]. Furthermore, our study showed that only 40% (n=40) of the interns reported receiving sufficient educational sessions about medication safety and prevention of MEs. Similarly, a multiregional study among healthcare practitioners (HCPs) in the KSA assessed their perception and knowledge about MEs and the reporting system and found that 54.8% (n=537) of the HCPs did not receive any training about the topic over a whole year [23]. This shows deficiencies in medication safety education are present from the level of junior healthcare students to senior HCPs. A systematic review assessing final-year medical students' competencies in prescribing medications discovered that final-year medical students did not have the skills to prescribe drugs safely [24]. This was further supported by a study investigating undergraduate nursing students' medication safety competence. In the study, they used a medication assessment tool that showed their level of competency was 29.6% (n=56) for juniors and 14.4% (n=27) for seniors [25]. This raises the question of whether our education and training systems efficiently address this paramount subject.

Furthermore, when assessing pharmacy students' knowledge and skills regarding patient safety in general, a study conducted in the KSA discovered that around half of the pharmacy students did not receive any courses regarding patient safety [26]. Deficiencies in nurses' knowledge were also noted; a study conducted in Rome found that when asked, a low percentage of 15.6% (n=16) of nurses judged their level of knowledge about the preparation and administration of IV medications as excellent. An overall 89.3% (n=92) considered improving their knowledge essential [27]. This highlights that post-graduate training should be continued regularly for healthcare professionals.

Study limitations

This is a cross-sectional survey-based study subjecting it to standard limitations of questionnaire-based studies such as recall bias, communication barriers, and social desirability bias. Most of the responses received were from interns in Riyadh city (83%, n=83), with the minority being from Khobar, Dammam, and Alkharj. This can make the data less representative of the Saudi population.

## Conclusions

Medication errors pose a serious threat to the well-being of patients. The research objective was to evaluate the knowledge and attitudes toward medication errors (ME) among healthcare interns and to identify the gaps in the current curricula regarding medication safety and the reporting system. This study concluded that medical, pharmacy, and nursing interns require more education and training to ensure safe medication use. This can be achieved by establishing a well-planned curriculum that can be incorporated into university programs. Moreover, additional studies need to be conducted about medication safety practices in Saudi Arabia among the undergraduate population, targeting a larger population size. In conclusion, addressing the deficiencies currently present in the knowledge and attitudes of interns can lead to better recognition and reporting of MEs, hence enhancing patient safety.

## Appendices

**Table 5:** The survey consists of 31 items and is divided into four sections: demographics, knowledge of MEs and the reporting system, participants’ attitudes, and universities’ current curricula on ME. ME: medication error

Section 1: Demographics	
Age	-
Gender	a. Male
	b. Female
Nationality	a. Saudi
	b. Non-Saudi
Marital status	a. Single
	b. Married
	a. Divorced
	b. Widowed
Employment status	a. Employed
	b. Not employed
University	a. Alfaisal University (AU)
	b. King Saud University (KSU)
	c. King Saud Bin Abdulaziz University for Health Sciences (KSAU-HS)
	d. Imam Muhammad Ibn Saud Islamic University (IMSIU)
	e. Almaarefa University
	f. Dar Al Uloom University
	g. Alfarabi Colleges
	h. Others
Intern in	a. College of Medicine
	b. College of Pharmacy
	c. College of Nursing
Section 2: Knowledge about medication errors and the reporting system	
I am aware of the definition of medication error.	a. Strongly disagree
	b. Disagree
	c. Neutral
	d. Agree
	e. Strongly agree
It is not my responsibility to report medication errors.	a. Strongly disagree
	b. Disagree
	c. Neutral
	d. Agree
	e. Strongly agree
If a medication error was prevented before reaching the patient, I would still report it.	a. Strongly disagree
	b. Disagree
	c. Neutral
	d. Agree
	e. Strongly agree
If a patient receives a medication that is not prescribed for them, I would not report it as medication error.	a. Strongly disagree
	b. Disagree
	c. Neutral
	d. Agree
	e. Strongly agree
I would report a medication error if the patient was harmed and required treatment.	a. Strongly disagree
	b. Disagree
	c. Neutral
	d. Agree
	e. Strongly agree
If a patient does not receive a medication as prescribed, I would report it as a medication error.	a. Strongly disagree
	b. Disagree
	c. Neutral
	d. Agree
	e. Strongly agree
If a patient is prescribed an inadequate dose of a medication, I would not report it as medication error.	a. Strongly disagree
	b. Disagree
	c. Neutral
	d. Agree
	e. Strongly agree
I am aware of the hospital Safety Reporting System (SRS).	a. Yes
	b. No
In your opinion, the main objective of medication error reporting system is to	a. Determine the safe use of the medication.
	b. Calculate incidence of the medication errors
	c. Identify factor that cause medication errors
	d. All of the above
Which of the following is considered as high-risk medication?	a. Anti-Infectives
	b. Potassium
	c. Insulins
	d. Narcotics
	e. All the above
The stages of medication errors are	a. Prescribing, transcription, dispensing, administration, patient education.
	b. Prescribing, transcription, independent double-check, administration, monitoring.
	c. Prescribing, transcription, dispensing, administration, monitoring.
	d. Prescribing, transcription, dispensing, clinical verification, monitoring.
Section 3: Attitude and barriers toward medication error reporting	
Medication errors do not need to be reported if they were detected before reaching the patient.	a. Strongly disagree
	b. Disagree
	c. Neutral
	d. Agree
	e. Strongly agree
The information I disclose when reporting a medication error will be confidential.	a. Strongly disagree
	b. Disagree
	c. Neutral
	d. Agree
	e. Strongly agree
It is not my responsibility to report a medication error cause by someone else.	a. Strongly disagree
	b. Disagree
	c. Neutral
	d. Agree
	e. Strongly agree
I do not need to report a medication error if I inform the patient involved.	a. Strongly disagree
	b. Disagree
	c. Neutral
	d. Agree
	e. Strongly agree
I prefer to educate people who make medication errors rather than report the errors.	a. Strongly disagree
	b. Disagree
	c. Neutral
	d. Agree
	e. Strongly agree
I fear of being blamed and punished if I report a medication error I made.	a. Strongly disagree
	b. Disagree
	c. Neutral
	d. Agree
	e. Strongly agree
Section 4: Assessing universities’ current curriculums on medication errors and safety	
My university’s curriculum includes sessions about medication safety and prevention of medication error.	a. Yes
	b. No
If answered yes to previous question, during which year did you receive those sessions? (enable choosing more than one option).	a. First year
	b. Second year
	c. Third year
	d. Fourth year
	e. Fifth year
	f. Internship
During which academic year do you think it is best to receive sessions about medication safety. (enable choosing more than one option).	a. First year
	b. Second year
	c. Third year
	d. Fourth year
	e. Fifth year
	f. Internship
The university curriculum includes enough sessions on medication safety and prevention of medication errors.	a. Strongly disagree
	b. Agree
	c. Neutral
	d. Agree
	e. Strongly agree
The university’s curriculum clearly explained to me the importance of medication error reporting.	a. Strongly disagree
	b. Disagree
	c. Neutral
	d. Agree
	e. Strongly Agree
The university’s curriculum explained how to report a medication error.	a. Strongly disagree
	b. Disagree
	c. Neutral
	d. Agree
	e. Strongly agree
The best way to learn about medication safety is through (enable choosing more than one option).	a. Traditional lectures
	b. PBLs (case scenarios)
	c. Real-life experience
	d. Simulation sessions
